# Schizandrin A Inhibits Microglia-Mediated Neuroninflammation through Inhibiting TRAF6-NF-κB and Jak2-Stat3 Signaling Pathways

**DOI:** 10.1371/journal.pone.0149991

**Published:** 2016-02-26

**Authors:** Fangjiao Song, Kewu Zeng, Lixi Liao, Qian Yu, Pengfei Tu, Xuemei Wang

**Affiliations:** 1 Research Studio of Integration of Traditional and Western Medicine, First Hospital, Peking University, Beijing, 100034, China; 2 State Key Laboratory of Natural and Biomimetic Drugs, School of Pharmaceutical Sciences, Peking University, Beijing, 100191, China; Universidade de São Paulo, BRAZIL

## Abstract

Microglial-mediated neuroinflammation has been established as playing a vital role in pathogenesis of neurodegenerative disorders. Thus, rational regulation of microglia functions to inhibit inflammation injury may be a logical and promising approach to neurodegenerative disease therapy. The purposes of the present study were to explore the neuroprotective effects and potential molecular mechanism of Schizandrin A (Sch A), a lignin compound isolated from *Schisandra chinesnesis*. Our observations showed that Sch A could significantly down-regulate the increased production of nitric oxide (NO), tumor necrosis factor (TNF)-α and interleukin (IL)-6 induced by lipopolysaccharide (LPS) both in BV-2 cells and primary microglia cells. Moreover, Sch A exerted obvious neuroprotective effects against inflammatory injury in neurons when exposed to microglia-conditioned medium. Investigations of the mechanism showed the anti-inflammatory effect of Sch A involved the inhibition of inducible nitric oxide synthase (iNOS) and cyclooxygenase 2 (COX-2) expression levels and inhibition of the LPS-induced TRAF6-IKKβ-NF-κB pathway. Furthermore, inhibition of Jak2-Stat3 pathway activation and Stat3 nuclear translocation also was observed. In conclusion, SchA can exert anti-inflammatory and neuroprotective effects by alleviating microglia-mediated neuroinflammation injury through inhibiting the TRAF6-IKKβ-NF-κB and Jak2-Stat3 signaling pathways.

## Introduction

Excessive neuroinflammation is increasingly considered as a vital factor in many neurodegenerative disorders, such as Alzheimer's disease (AD) and Parkinson's disease. The term neuroinflammation here refers to central nervous system (CNS)-specific, chronic microglia-mediated inflammation responses that contribute to the neurodestructive effects [1]. Microglia, which act as the first and main form of immune defense of the CNS, have dual functions in protection and injury processes under different conditions. In the homeostatic state, microglia constantly scan the CNS environment and scavenge damaged neurons, plaques and infectious agents before they can cause further damage to sensitive neural tissue. By contrast, once microglia are activated by factors released from injured cells, they will migrate to the infected or damaged site and then engage in degenerative and regenerative processes. Overactivated microglia can secrete a wide range of inflammatory factors and neurotoxic compounds, including nitric oxide (NO), tumor necrosis factor (TNF)-α, interleukin (IL)-6 and reactive oxygen species (ROS), which are deleterious to bystander neurons and impact their processes. Microglia-mediated inflammation can cause secondary injury following the development of neurodegenerative disease and result in neuronal cell death. Thus, the inhibition of overactive microglia represents a potentially effective treatment strategy for neurodegenerative disease.

Accumulating lines of evidence indicate that Toll-like receptor 4 (TLR4) can detect lipopolysaccharide (LPS) from Gram-negative bacteria and is central to the activation of microglia via various inflammatory signaling cascades [2]. Nuclear factor-κB (NF-κB) and Janus kinase/signal transducer and activator of transcription (Jak/Stat) pathways are well-known pathways in the regulation of inflammatory responses. NF-κB, as a transcription factor, plays a vital role in regulating the immune response to infection by controlling inflammatory gene expression and cytokine production [3–5]. Jak-Stat pathways have been reported to be involved in microglia-mediated inflammation responses induced by LPS and endogenous production of interferon (IFN)-γ and IL-6 [6–7].

Long-term use of non-steroidal anti-inflammatory drugs (NSAIDs) has been associated with decreased risk of developing neurodegenerative disease. However, due to the high risk of adverse drug reactions, such as damage to the gastrointestinal tract, the use of NSAIDs is not always well-accepted. Therefore, safer, better tolerated and more powerful anti-inflammation drugs for neurodegenerative disease are greatly desired. Currently, studies of neuroprotective agents from natural sources constitute an active area of research. For example, Schizandrin A (Sch A) is a bioactive lignin compound with potential neuroprotective effects that was isolated from the fruit of *Schisandra chinensis* (Turcz) Baill, which long has been used in traditional Chinese medicine to treat spontaneous sweating, chronic asthma, insomnia and amnesia. A previous study indicated that Sch A could protect primary cortical neurons from L-glutamate-induced neurotoxicity [8]. Moreover, Sch A was found to significantly improve the viability of primary cortical cells in an oxygen-glucose deprivation/reoxygenation model by reducing the intracellular calcium concentration and lactate dehydrogenase release [9]. These studies provided evidence to support the ability of Sch A to protect neuronal cells against various neurotoxicity injuries; however, few studies have focused on investigating its anti-neuroinflammatory effects. In our study, we observed that Sch A could down-regulate NO, IL-6 and TNF-α production in LPS-induced BV-2 cells, a classical model for neuroinflammation. However, the anti-neuroninflammatory mechanism remains obscure. Therefore, the present study was designed to further validate the anti-inflammatory potential of Sch A and elucidate the potential mechanism involved.

## Material and Methods

### Ethics Statement

The new born ICR mouse pups were sacrificed by decapitation. All procedures of animal experimentation were conducted in accordance with “the Guide for the Care and Use of Laboratory Animals” of the National Institutes of Health and Peking University Guidelines of Animal Care and Use. The experimental protocols were approved by the Institutional Animal Care and Use Committee of Peking University Health Science Center. Animals were housed under controlled conditions of temperature (22±1°C), humidity (50%) and 12 h light-dark cycles. All efforts were made to ameliorate the welfare and minimize animal suffering.

### Materials

**Fig 1:**
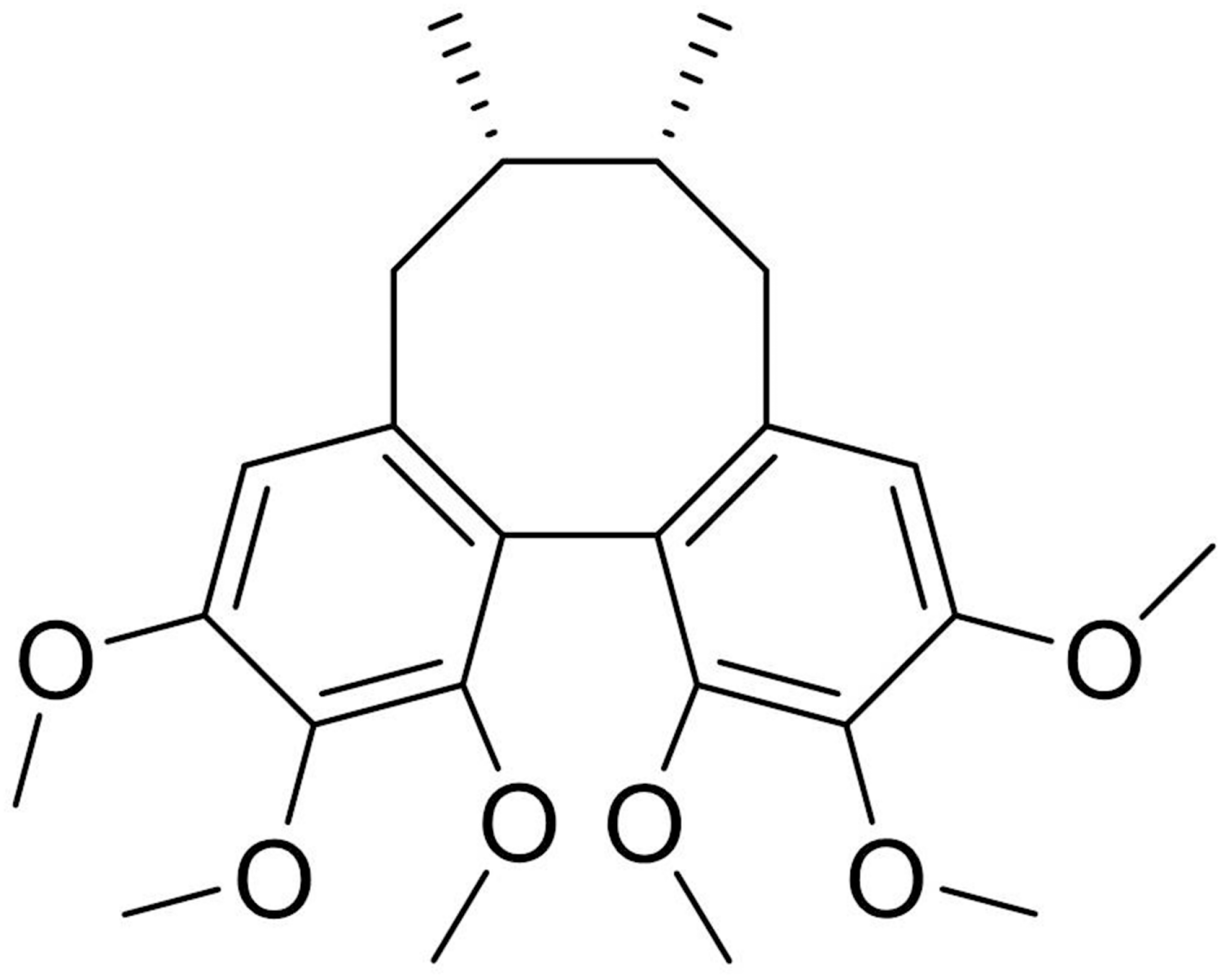
Chemical structure of Sch A.

Sch A (C_24_H_28_O_7_) was obtained from the National Institutes for Food and Drug Control (Beijing, China), and its structure is shown in Fig 1. High-performance liquid chromatography showed the purity of Sch A was greater than 98%. Dulbecco’s modified Eagle’s minimum essential medium (DMEM), fetal bovine serum (FBS), antibiotics and trypsin were obtained from Hyclone (Logan, UT, USA). Neurobasal medium and serum-free B27 supplement were purchased from Invitrogen (Carlsbad, CA, USA). 3-[4,5-dimethylthiazol-2-yl] 2,5-diphenyltetrazolium bromide (MTT), LPS (*Escherichia coli*, serotype 055:B5), Hoechst 33258 and crystal violet were purchased from Sigma Chemical Co. (St Louis, MO, USA). Dimethylsulfoxide (DMSO) was from Beijing Chemical Works (Beijing, China). The NO assay kit was obtained from Nanjing Jiancheng Bioengineering Institute (Jiangsu, China). TNF-α and IL-6 ELISA kits were obtained from ExCell Bio Company (Shanghai, China). Easy pure PCR purification kit was from TransGen Biotech Inc., (Beijing, China). BioepitopeR protein A+G agarose IP sample was obtained from Bioworld Technology Inc. (St. Louis Park, MN, USA). Sheared salmon sperm DNA was purchase from Biological Reagent Technology Inc. (Beijing, China). All antibodies were purchased from Cell Signaling Technology (Beverly, MA, USA). Western chemiluminescent horseradish peroxidase (HRP) substrate was from Thermo Fisher Scientific. (Waltham, MA, USA).

### Cell culture

The mouse BV-2 (microglial) and RAW264.7 (macrophage) cell lines were obtained from the cell center of the Chinese Academy of Medical Sciences (Beijing, China). Cells were maintained in high-glucose DMEM medium containing 10% (*v/v*) heat-inactivated FBS, 100 U/ml penicillin and 100 μg/ml streptomycin at 37°C in a humidified atmosphere with 5% CO_2_.

### Primary microglial culture

Primary microglia were isolated from the frontal cortices of newborn ICR mouse pups. Briefly, the cortex tissues were resected, followed by removal of the meninges aseptically. The tissues were then cut into approximately 1 mm^3^ fragments and digested in 0.2% trypsin for 20 min at 37°C. The digested cells were seeded in a poly-L-lysine-coated 75 cm^2^ flask and incubated with DMEM/F12 containing 10% FBS for 2 weeks as mixed glial cells. After the mixed glial cultures were completely confluent, the cells were washed with DMEM/F12 to eliminate serum and incubated with trypsin (0.25%): DMEM/F12 = 1:3 at 37°C until the upper intact layer was detached. The medium containing the layer of detached cells which included mainly astroglia was aspirated, while the remaining cells were mostly microglia. Isolated microglia were recovered by incubating with 0.25% trypsin for 5 min followed by vigorous pipetting. The loosen microglia seeded in 48-well culture dishes were used in the following experiments.

### Treatment of neuronal cultures with microglia-conditioned medium

Primary cortical neurons were obtained from dissociated embryonic (E17-18) cortices of ICR mouse fetuses. In brief, after removing the meninges, the cortices were cut into approximately 1 mm^3^ pieces and digested in 0.2% trypsin at 37°C for 20 min. The neurons were seeded onto poly-L-lysine coated culture plates in DMEM containing 10% FBS. The medium was replaced by neurobasal medium containing B27 after 4 h of culture. The cells were exposed to microglia-conditioned medium after 7 days of culture.

After BV-2 cells were treated with LPS (1 μg/ml) with or without Sch A (10, 20 and 50 μM) for 6 h, the supernatant containing drugs were exchanged with neurobasal medium containing B27. The activated BV-2 cells continued to secrete inflammation mediators into the neurobasal medium during the following 24 h of culture. The conditioned medium was then incubated with isolated neurons to establish a microglia-neuron co-culture. After 24 h of culture, the neurons were used in subsequent assays.

### Cell viability assay

Cell viability was evaluated by the MTT assay. After treatment, MTT (5 mg/ml) was added to the culture medium and incubated at 37°C for 2 h. The formazan crystals formed were dissolved in DMSO, and the absorbance was read at 570 nm. Cell viability was expressed as the percentage of the control.

### NO assay

The accumulation of NO in the culture medium was determined by the Griess method. After treatment with LPS (1 μg/ml) with or without Sch A (10, 20 and 50 μM) for 24 h, the cell culture supernatants were collected and reacted with equal amounts of Griess reagent (1% sulfanilamide/0.1% naphthylethylene diamine dihydrochloride/2% phosphoric acid). Subsequently, the optical density was read at 540 nm, and sodium nitrite was used for a standard curve in the assay.

### ELISA for TNF-α, IL-6 and IL-10

After treatment, the supernatants were collected for the subsequent proinflammatory mediator assay. TNF-α, IL-6 and IL-10 concentrations were measured using commercial ELISA kits according to the manufacturer's protocol.

### Western blot analysis

After treatment, BV-2 cells were collected and homogenized with ice-cold RIPA buffer (50 mM Tris-HCl, 300 mM NaCl, 0.5% TritonX-100, 5 mM EDTA, cocktail protease inhibitor) for 20 min. The cell lysate was centrifuged at 13,000 rpm for 20 min at 4°C. Nuclear and cytoplasmic proteins were prepared using the Nuclear Extraction kit (KeyGEN Biotech Inc., Nanjing, China) according to the manufacturer's protocol. Equal amounts of proteins were separated using SDS-polyacrylamide gel electrophoresis and transferred to polyvinylidene fluoride membranes. The membranes were blocked by 5% skim milk and incubated with primary antibodies overnight at 4°C. After incubation with a HRP-labeled secondary antibody at room temperature for 1 h, the membranes were developed with enhanced chemiluminescence (ECL) and visualized using a Digital Imaging System (Gel Logic 2200 Pro, Kodak, Tokyo, Japan).

### Chromatin immunoprecipitation (ChIP) assays

ChIP assays were performed as described previously with some modifications [10]. Briefly, BV-2 cells were treated with LPS (1 μg/ml) with or without Sch A (50 μM) for 2 h. 1% of formaldehyde was added to the culture medium, and after incubation at 37°C for 10 minutes, cells were rinsed twice with cold PBS and lysed for 10 minutes on ice with lysis buffer (1% SDS, 5 mM EDTA, 50 mM Tris-HCl, pH 8.1, cocktail protease inhibitor). After sonication, 20% of total supernatant was used as DNA input control. The remaining lysates were diluted 10-fold with ChIP dilution buffer (1% Triton X-100, 2 mM EDTA, 150 mM NaCl, 20 mM Tris-HCl, pH 8.1, protease inhibitor cocktail) followed by precleared with 2μg sheared salmon sperm DNA, 5μg normal IgG, and 50μl protein A+G agarose beads for 2 h at 4°C. The precleared lysates were immunoprecipitated with NF-κB p65 and normal Rabbit IgG antibodies overnight at 4°C. Precipitates were collected with protein A+G Plus-agarose beads and washed sequentially for 10min each in TSE I (150 mM NaCl, 20 mM Tris-HCl, pH 8.1, 2 mM EDTA, 1% Triton X-100 and 0.1% SDS), TSE II (500 mM NaCl, 20 mM Tris-Cl, pH 8.1, 2 mM EDTA, 1% Triton X-100, and 0.1% SDS), buffer III (1 mM EDTA, 1% deoxycholate, 1% Nonidet P-40 and 0.25 M LiCl, 10 mM Tris-HCl, pH 8.1) and three times in TE buffer (1 mM EDTA, 10 mM Tris-Cl, pH 8.0). The immunoprecipitated complex was then eluted with 1% SDS and 0.1 M NaHCO_3_ at room temperature for 10 minutes. Cross-linking of protein-DNA complexes was reversed at 65°C for 6 h. DNA was extracted with the Easy pure PCR purification kit. Purified DNA were used as the template for PCR under the following condition: 94°C for 5 min followed by 30 cycles of 94°C for 0.5 min, 54°C for 0.5 min, 72°C for 1 min and a final extension at 72°C for 10 min. The sequences of primers for iNOS promoter are as follows: forward: 5’-AACTATTGAGGCCACACACT -3’ and reverse: 5’-AGTGTTAGGGGAAAAGGAAC -3’. Each sample was analyzed three times. Relative gene level was normalized to input control and was calculated by the following formula: Relative level of gene (folds of control) = 2^-ΔΔCT^

### Immunofluorescence assay

After treatment, BV-2 cells or primary neurons (seeded on glass coverslips) were fixed with cold 4% paraformaldehyde for 20 min at room temperature, permeabilized (0.5% TritonX100) for 30 min and blocked (5% BSA in PBST) for 1 h at room temperature. The cells were then stained with primary antibodies overnight at 4°C, followed by secondary antibodies conjugated to Alexa 488 (1:1000) for 1 h at room temperature. The cells were further stained with 4’, 6-diamidino-2-phenyl indole (DAPI, 5 mg/ml in PBS) for 20 min at 37°C. After washing and sealing, the stained cells were imaged using a fluorescence microscope (IX73, Olympus, Japan).

### Crystal violet staining assay

After the primary neurons were treated with conditioned medium for 24 h, morphological assessment of apoptotic neurons was performed by crystal violet staining. In brief, cells were washed with PBS and fixed in cold 4% paraformaldehyde for 20 min at room temperature. The crystal violet (0.5%) solution was added to the cells for incubation for 30 min at room temperature. Afterwards, cells were washed with PBS, and photographs were taken with an optical microscope (IX73, Olympus, Japan).

### Hoechst 33258 apoptosis assay

After treatment with conditioned medium for 24 h, the neurons were fixed with cold 4% paraformaldehyde for 20 min, followed by staining with Hoechst 33258 solution for 10 min at 37°C. The cells were then washed three times with PBS, and images were observed under a fluorescence microscope (IX73, Olympus, Japan).

### Statistical analysis

Data are presented as the mean ± standard deviation (S.D.) from at least three independent experiments. Statistical analysis between different groups was performed using one-way analysis of variance (ANOVA) with Tukey's multiple comparison post-test with SPSS version 16.0. *P* < 0.05 was considered to be statistically significant.

## Results

### Sch A downregulates inflammatory mediators in LPS-induced BV-2 cells

**Fig 2:**
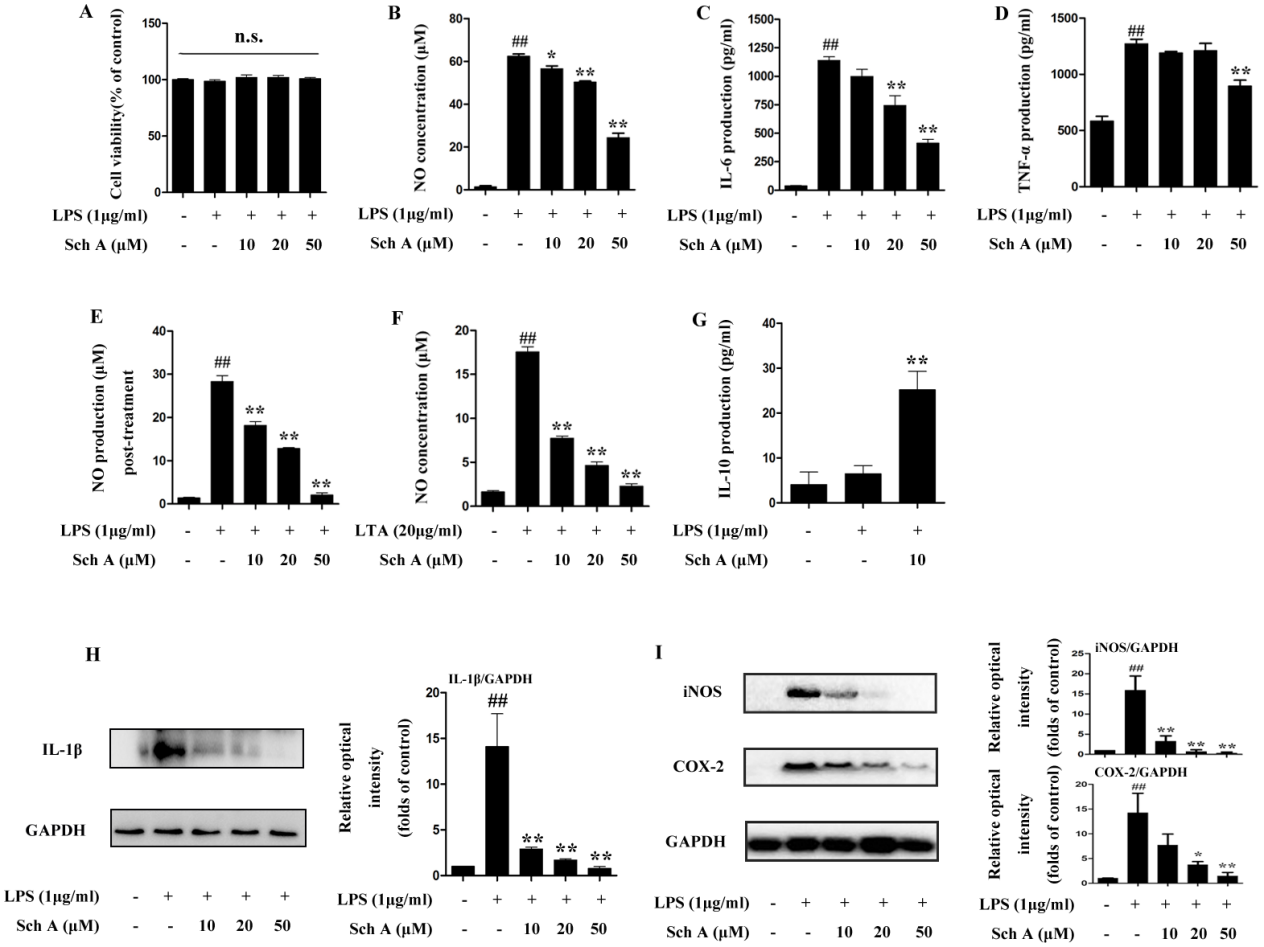
Sch A inhibition of inflammation response by down-regulating expression of inflammatory mediators. (A) BV-2 cells were treated with LPS (1 μg/ml) with or without Sch A (10, 20 and 50 μM) for 24 h, and then cell viability was determined by the MTT assay. (B) BV-2 cells were treated with LPS (1 μg/ml) with or without Sch A (10, 20 and 50 μM) for 24 h, and then NO production was quantified by an assay kit. (C) BV-2 cells were treated with LPS (1 μg/ml) with or without Sch A (10, 20 and 50 μM) for 8 h, followed analysis of IL-6 expression. (D) BV-2 cells were treated with LPS (1 μg/ml) with or without Sch A (10, 20 and 50 μM) for 4 h, followed analysis of TNF-α expression. (E) BV-2 cells were treated with LPS (1 μg/ml) for 2 h, and then incubated with Sch A (10, 20 and 50 μM) for 24 h. NO production was tested by an assay kit. (F) BV-2 cells were treated with LTA (20 μg/ml) with or without Sch A (10, 20 and 50 μM) for 24 h, followed by analysis of NO production. (G) BV-2 cells were treated with LPS (1 μg/ml) with or without Sch A (10μM) for 24 h, followed by analysis of IL-10 production. (H) After BV-2 cells were treated with LPS (1 μg/ml) with or without Sch A (10, 20 and 50 μM) for 24 h, western blot analysis of IL-1β expression was done. (I) BV-2 cells were treated with LPS (1 μg/ml) with or without Sch A (10, 20 and 50 μM) for 24 h, and then iNOS and COX-2 protein levels were analyzed by Western blot. All data are shown as the mean ± S.D. from independent experiments performed in triplicate. ^##^*P* < 0.01 relative to control group; *P < 0.05, **P < 0.01, relative to LPS group.

First, we examined the cytotoxicity of Sch A (10, 20 and 50 μM) by using the MTT assay and found that even the highest concentration of Sch A did not affect cell viability (Fig 2A). As shown in Fig 2B–2D and 2H, significant increases in expression of NO, IL-6, TNF-α and IL-1β resulting from LPS stimulation in BV-2 cells were suppressed by Sch A in a concentration-dependent manner. Moreover, when BV-2 cells were pre-treated with LPS for 2 h and then incubated with Sch A for 24 h, the increased production of NO induced by LPS was decreased by Sch A in a dose-dependent manner (Fig 2E). BV-2 cells were stimulated to produce excessive inflammatory responses by secreting abundant NO when treated by lipoteichoic acid (LTA, 20 μg/ml), a cell wall component of Gram-positive bacteria. As shown in Fig 2F, the inflammatory reaction induced by LTA in BV-2 cells was nearly completely reversed by Sch A treatment. IL-10 which plays an immunosuppressive role in inflammatory responses could be enhanced by Sch A (10 μM, Fig 2G). However, when we did the research of Sch A effect on LPS induced RAW264.7 cells, which play a vital role in the peripheral immune system, and found Sch A could only down-regulate the secretion of NO but has no effect on IL-6 and TNF-α production. (S1 Fig). From the above results, we concluded that Sch A could exert a relatively sepecific anti-neuroninflammatory effects in BV-2 cells by inhibiting the production of proinflammatory mediators in different pathological conditions. iNOS and COX-2 are two crucial enzymes which are central to the inflammatory response. Using Western blot analysis, we tested the effect of Sch A on iNOS and COX-2 expression in LPS-induced BV-2 cells, The results showed that Sch A could significantly decrease both iNOS and COX-2 expression levels in a dose-dependent manner (Fig 2I).

### Sch A inhibits primary microglia activation induced by LPS and protects neurons from microglia-mediated inflammatory injury

**Fig 3:**
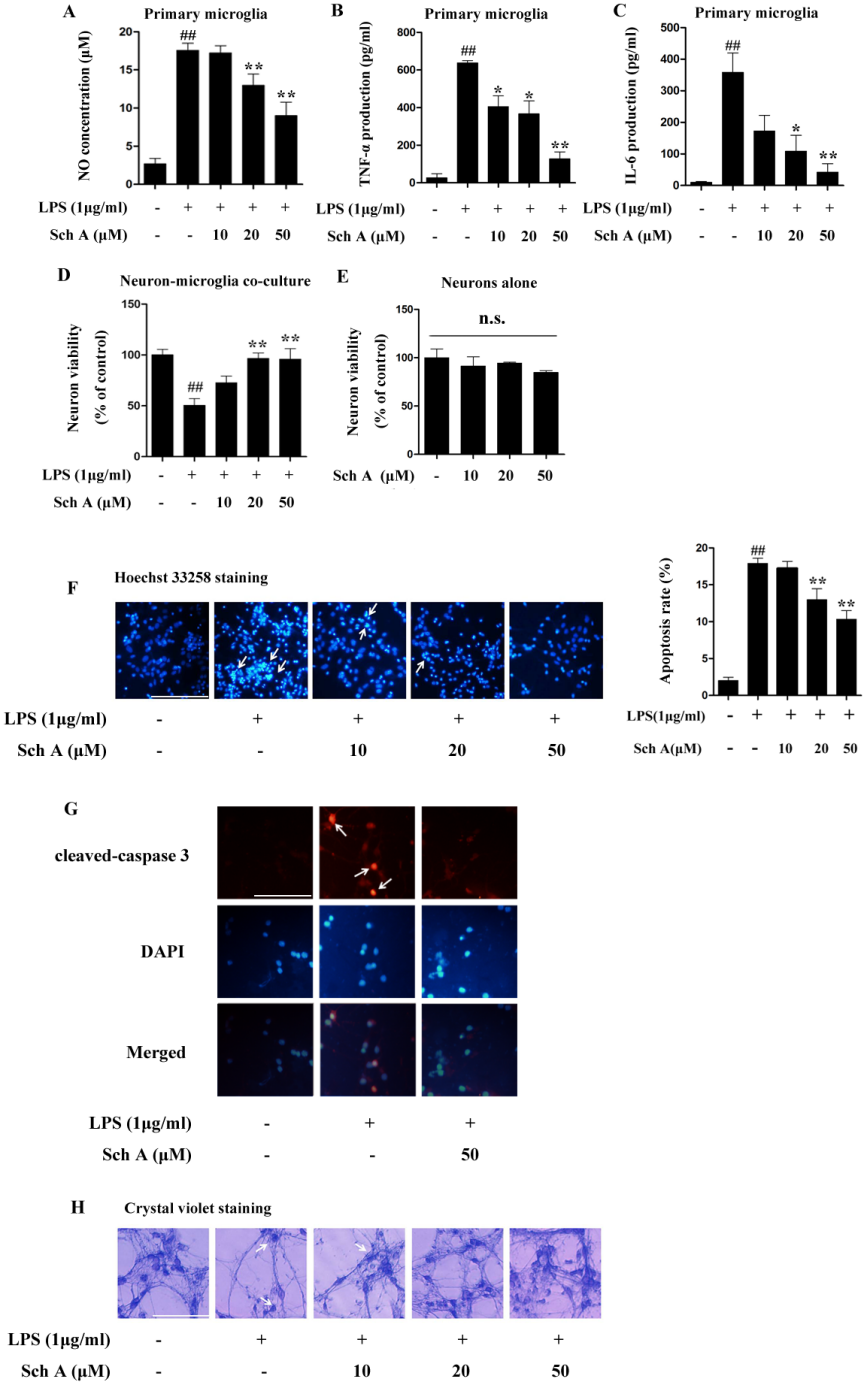
Sch A inhibition of primary microglia activation induced by LPS and protection of neurons from microglia-mediated inflammatory injury. (A) Primary microglia cells were treated with LPS (1 μg/ml) with or without Sch A (10, 20 and 50 μM) for 24 h, followed by analysis of NO production. (B) Primary microglia cells were treated with LPS (1 μg/ml) with or without Sch A (10, 20 and 50 μM) for 4 h, and then TNF-α expression was analyzed by ELISA. (C) Primary microglia cells were treated with LPS (1 μg/ml) with or without Sch A (10, 20 and 50 μM) for 8 h, and then IL-6 expression was analyzed by ELISA. (D) Primary neuron viability was tested using the MTT assay after treatment with conditioned medium and induction by LPS (1 μg/ml) with or without Sch A (10, 20 and 50 μM) for 24 h. (E) After primary neurons were treated with or without Sch A (10, 20 and 50 μM) for 24 h, cell viability was determined by the MTT assay. (F) After primary neurons were incubated with conditioned medium and stimulated by LPS (1 μg/ml) with or without Sch A (10, 20 and 50 μM) for 24 h, Hoechst33258 staining was performed to assess cellular apoptosis. Typical apoptotic cells are marked by arrows (bar = 100 μm). Apoptosis rate = number of positive cells/total number of cells × 100% (G) After primary neurons were incubated with conditioned medium and induced by LPS (1 μg/ml) with or without Sch A (50 μM) for 24 h, cleaved caspase 3 was detected by immunofluorescence. Red fluorescence represents cleaved caspase 3, and blue fluorescence represents nuclear DAPI staining (bar = 100 μm). (H) After primary neurons were incubated with conditioned medium and induced by LPS (1 μg/ml) with or without Sch A (10, 20 and 50 μM) for 24 h, crystal violet staining was performed to observe changes in morphology. Typical apoptotic cell are marked by arrows (bar = 100 μm). All data are shown as the mean ± S.D. from independent experiments performed in triplicate. ^##^*P* < 0.01 relative to control group; *P < 0.05, **P < 0.01 relative to LPS group.

We explored the anti-inflammatory effect of Sch A in primary cortical microglia induced by LPS. Primary microglia were treated with LPS (1 μg/ml) with or without Sch A (10, 20 and 50 μM) for 8 h (IL-6 assay), 4 h (TNF-α assay) or 24 h (NO assay), and then inflammation mediators were quantified by using the appropriate assay kit. As shown in Fig 3A–3C, treatment with Sch A could significantly decrease the production of NO, TNF-α and IL-6 stimulated by LPS. In the following experiments, we tested the neuronprotective effect of Sch A in neurons exposed to microglia-conditioned medium. Tests of neuronal viability clearly showed that LPS treatment resulted in death of neurons through microglia-mediated inflammation injury; however, treatment with Sch A significantly suppressed the neuronal death in a dose-dependent manner (Fig 3D). Next, we tested the effect of only Sch A (10, 20 and 50 μM) and found that it had no effect on neuron viability, demonstrating that the neuroprotective effect of Sch A may be realized by inhibiting the microglia-mediated neurovirulent inflammatory response (Fig 3E). Furthermore, Hoechst 33258 staining and cleaved caspase 3 immunofluorescence showed that LPS treatment led to obvious neuronal apoptosis; however, the apoptosis was largely reversed by treatment with Sch A (Fig 3F and 3G). Analysis of cell morphology by crystal violet staining clearly showed that neurons suffered inflammation-related injury presented as neurite loss and cleavage when treated with LPS-induced conditioned medium, and Sch A significantly alleviated the neuronal injury (Fig 3H).

#### Sch A inhibits NF-κB pathway activation in LPS-induced BV-2 cells

**Fig 4:**
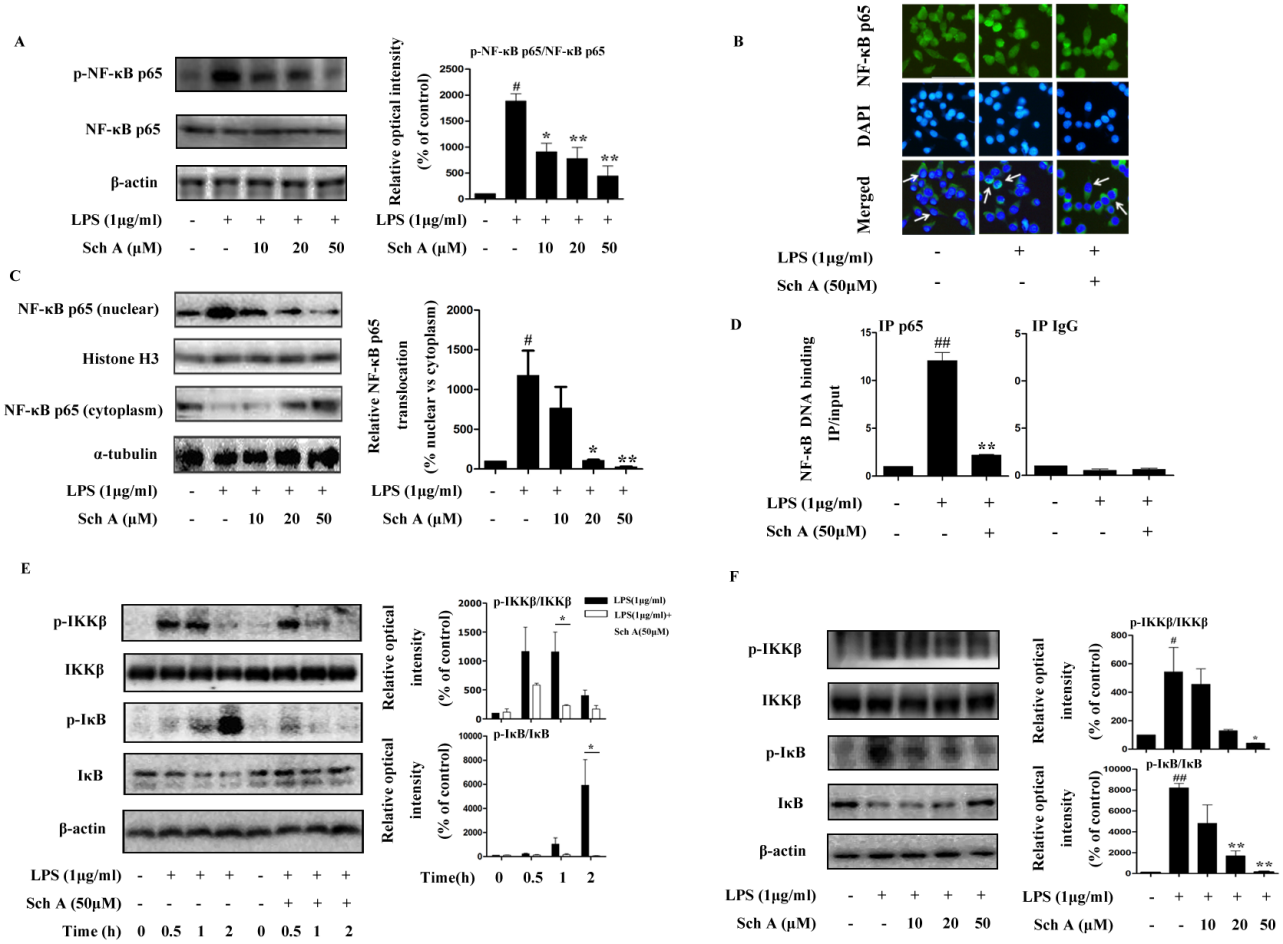
Sch A inhibition of NF-κB pathway activation in LPS-induced BV-2 cells. (A) BV-2 cells were treated with LPS (1 μg/ml) with or without Sch A (10, 20 and 50 μM) for 2 h, followed by analysis of NF-κB p65 phosphorylation by Western blot. (B) BV-2 cells were treated with LPS (1 μg/ml) with or without Sch A (50 μM) for 2 h, followed by detection of the NF-κB p65 subunit translocation by immunocytochemistry. Green fluorescence represents the NF-κB p65 subunit, and blue fluorescence represents nuclear DAPI staining (bar = 50 μm). (C) After BV-2 cells were treated with LPS (1 μg/ml) with or without Sch A (10, 20 and 50 μM) for 2 h, NF-κB p65 levels in the nucleus and cytoplasm were determined by Western blot. Histone H3 and α-tubulin were used as endogenous controls for nuclear and cytoplasmic proteins, respectively. (D) After BV-2 cells were treated with LPS (1 μg/ml) with or without Sch A (50 μM) for 2h. Cell lysates were prepared for chromatin immunoprecipitation for NF-κB p65, samples were amplified by quantitative PCR with primers for the promoter of iNOS. (E) BV-2 cells were treated with LPS (1 μg/ml) with or without Sch A (50 μM) for 0, 0.5, 1 and 2 h. The phosphorylated and total IKKβ and IκB proteins at different time points were determined by Western blot. (F) After BV-2 cells were treated with LPS (1 μg/ml) with or without Sch A (10, 20 and 50 μM) for 0.5 h (p-IKKβ assay) or 1 h (p-IκB assay), the phosphorylated and total IKKβ and IκB proteins were determined by Western blot. All data are shown as the mean ± S.D. from independent experiments performed in triplicate. ^#^*P* < 0.05, ^##^*P* < 0.01 relative to control group; *P < 0.05, **P < 0.01 relative to LPS group.

NF-κB serves as a vital mediator of the inflammatory response, and its activation is involved in the early stage of neurodegenerative diseases [11]. The above data showed that Sch A could suppress the expression of pro-inflammatory mediators and cytokines regulated by NF-κB. Therefore, we further investigated the role of Sch A in the activation of the NF-κB pathway. First, we found that Sch A could dose-dependently suppress the increased phosphorylation levels of NF-κB p65 stimulated by LPS treatment (Fig 4A). The activation of NF-κB by phosphorylation, triggered its nuclear translocation and initiation of inflammatory gene expression. Analysis of the NF-κB p65 subcellular localization by an immunofluorescence assay revealed that the accumulation of NF-κB p65 in the nucleus induced by LPS could be alleviated by Sch A (50 μM) treatment (Fig 4B). Moreover, we quantified nuclear and cytosolic fractions of the NF-κB p65 subunit by Western blotting and found that the sharply increased nuclear translocation of NF-κB p65 stimulated by LPS could be blocked by Sch A treatment in a dose-dependent manner (Fig 4C). After nuclear translocation, NF-κB p65 could bind to target DNA and start the transcription, but from the ChIP assay, we found this phenomenon was reversed by Sch A (50 μM) treatment (Fig 4D).

IKKβ and IκB are two important modulators of the upstream NF-κB signal transduction cascade. IKKβ acts as a protein subunit of IκB kinase, and IκB retains NF-κB in an inactive state in the cytoplasm. We treated BV-2 cells with LPS (1 μg/ml) with or without Sch A (50 μM) for 0, 0.5, 1 and 2 h and determined phosphorylation levels and total protein expression of IKKβ and IκB by Western blotting. The results indicated that Sch A significantly decreased the phosphorylated levels of IKKβ and IκB at the 0.5 h and 1 h time points, respectively (Fig 4E). Moreover, Sch A dose-dependently decreased IKKβ and IκB phosphorylation levels when treated with LPS (1 μg/ml) for 0.5 h and 1 h, respectively (Fig 4F). The results above indicated that Sch A could effectively suppress the activity of the LPS-induced IKKβ-IκB-NF-κB signaling pathway.

#### Sch A inhibits TRAF6 expression of NF-κB pathway in LPS-induced BV-2 cells

**Fig 5:**
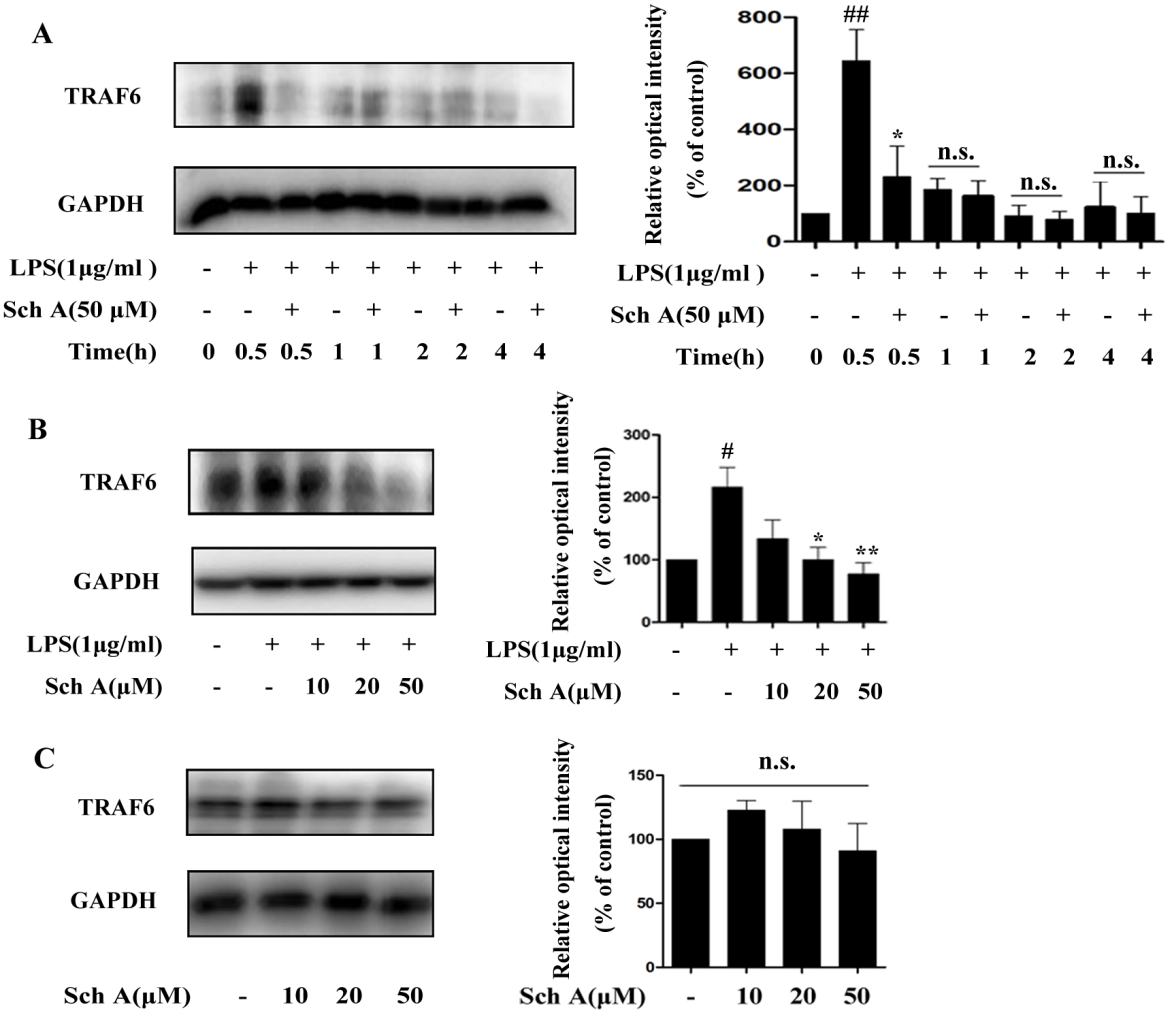
Sch A inhibition of TRAF6 expression of NF-κB pathway in LPS-induced BV-2 cells. (A) BV-2 cells were treated with LPS (1 μg/ml) with or without Sch A (50 μM) for 0, 0.5, 1, 2 and 4 h, and then TRAF6 protein expression was measured by Western blot. (B) BV-2 cells were treated with LPS (1 μg/ml) with or without Sch A (10, 20 and 50 μM) for 0.5 h, and then TRAF6 expression was measured by Western blot. (C) BV-2 cells were treated with Sch A (10, 20 and 50 μM) for 0.5 h, and then TRAF6 was measured by Western blot. All data are shown as the mean ± S.D. from independent experiments performed in triplicate. ^#^*P* < 0.05, ^##^*P* < 0.01 relative to control group; *P < 0.05, **P < 0.01 relative to LPS group.

TRAF6 protein is a signal transducer in the NF-κB pathway which can activate IKKβ in response to signals from receptors. First, we tested the effect of Sch A on TRAF6 expression induced by LPS at different time points (0, 0.5, 1, 2 and 4 h). TRAF6 expression was markedly increased upon treatment with LPS for 0.5 h, and Sch A could significantly inhibit its expression (Fig 5A). Further analysis showed that Sch A could suppress TRAF6 expression in a dose-dependent manner when BV-2 cells were treated with LPS (1 μg/ml) with or without Sch A (10, 20 and 50 μM) for 0.5 h (Fig 5B). Moreover, Sch A only treatment did not have an effect on TRAF6 expression (Fig 5C). The results indicated that Sch A could reverse the effect of LPS on TRAF6 expression.

#### Sch A inhibits Jak2-Stat3 pathway activation in LPS-induced BV-2 cells

**Fig 6:**
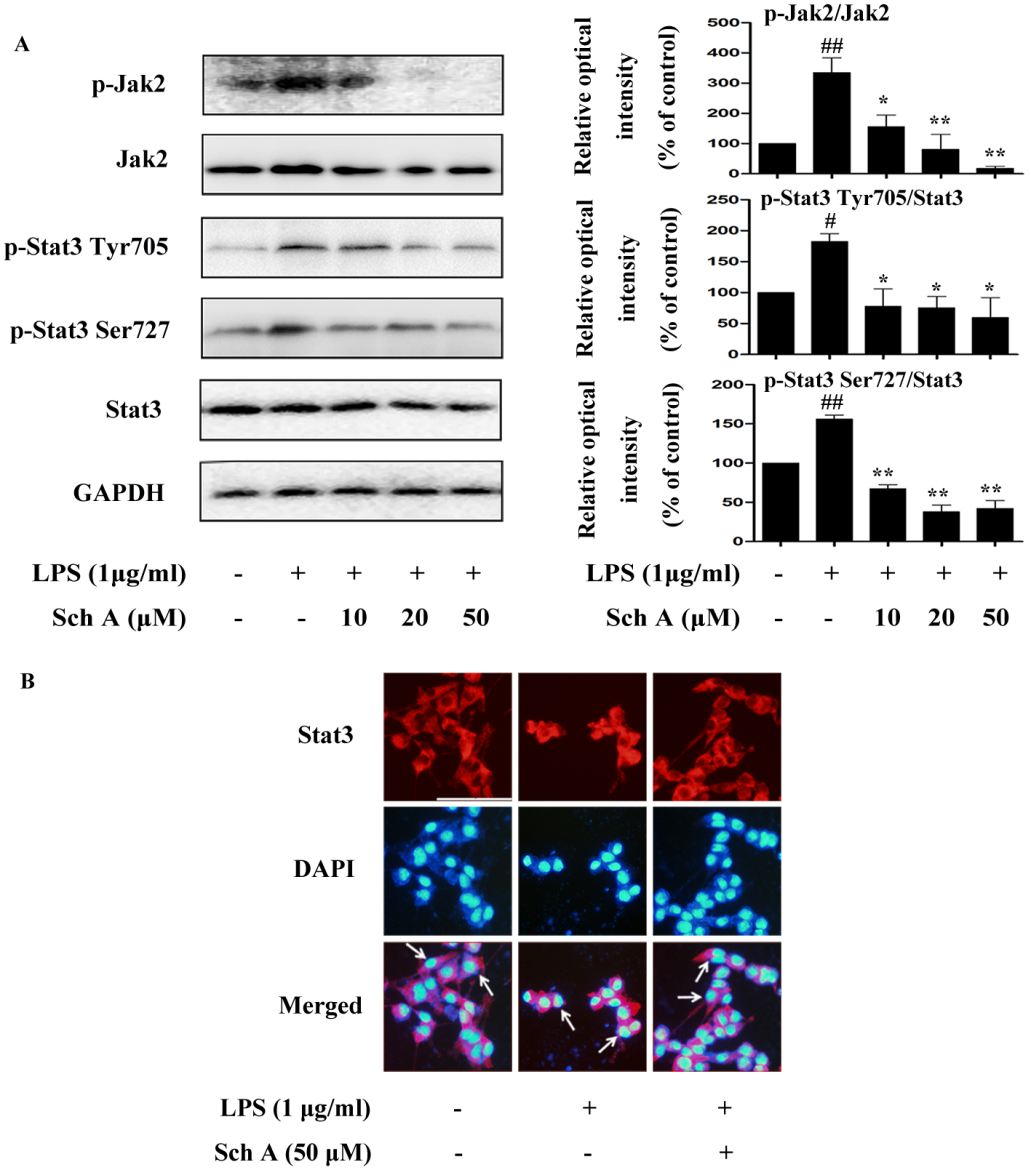
Sch A inhibition of Jak2-Stat3 pathway activation in LPS-induced BV-2 cells. (A) BV-2 cells were treated with LPS (1 μg/ml) with or without Sch A (10, 20 and 50 μM) for 0.5 h, Western blot analysis was performed to detect phosphorylated and total protein levels of Jak2, Stat3 Tyr705 and Stat3 Ser727. (B) BV-2 cells were treated with LPS (1 μg/ml) with or without Sch A (50 μM) for 2 h, and Stat3 nuclear translocation was detected by immunocytochemistry. Red fluorescence represents Stat3, and blue fluorescence represents nuclear DAPI staining (bar = 100 μm). All data are shown as the mean ± S.D. from independent experiments performed in triplicate. ^#^*P* < 0.05, ^##^*P* < 0.01 relative to control group; *P < 0.05, **P < 0.01 relative to LPS group.

The Jak-Stat cascade is an essential signaling pathway in inflammatory activities and immune responses. Jak2 can transfer LPS-induced signals to downstream molecules and activate Stat3 phosphorylation. The activated Stat3 then translocates from the cytoplasm into the nucleus to initiate expression of proinflammatory genes. Therefore, we determined the effects of Sch A on the Jak2-Stat3 signaling pathway. The results showed that the markedly enhanced phosphorylation levels of Jak2 and Stat3 (Tyr 705 and Ser 727) induced by LPS were significantly suppressed by Sch A treatment in a dose-dependent manner (Fig 6A). As shown in Fig 6B, LPS significantly induced the translocation of Stat3 from the cytoplasm to nucleus in BV-2 cells; however, Sch A effectively reversed this process by increasing Stat3 in the cytoplasm. From the above data, we concluded that Sch A may achieve an anti-inflammatory effect by inhibiting Jak2 and Stat3 phosphorylation and further blocking the nuclear translocation of Stat3.

## Discussion

Accumulating experimental evidence and autopsy observations have indicated that neuroinflammation is the primary mechanism associated with AD pathogenesis. Microglia, as resident immune cells and microsensors which fulfill a range of different tasks, are mainly involved in the inflammatory response and in maintaining homeostasis within the CNS [12]. Microglia can become over-reactive when stimulated by damaged neurons and other insults. The over-activated microglia which can produce various pro-inflammatory mediators are believed to contribute to and even aggravate neuronal injury in neurodegenerative diseases [13–14]. Therefore, the inhibition of the extent and duration of inflammation mediated by microglia may be a promising and logical therapeutic approach for neurodegenerative diseases.

Overexpression of inflammation-related mediators and cytokines generated from activated microglia can result in inflammatory disorders [15]. In our studies, we found that Sch A could clearly suppress the increased production of NO, TNF-α, IL-6 and IL-1β induced by LPS in BV-2 cells. In addition, Sch A markly downregulated the expression levels of iNOS and COX-2, two important enzymes in the inflammatory response. The anti-inflammation effect of Sch A could also be manifested in primary microglia by down-regulating increased NO, TNF-α, and IL-6 production induced by LPS. Moreover, based on results of the apoptosis and morphological assays, Sch A could protect neurons from inflammation-related injury induced by LPS and improve neuron cell viability. Together, the results indicate that Sch A may serve as a potential anti-inflammatory agent and is worthy of further study.

To explore the potential mechanisms of the anti-inflammatory effect of Sch A, we focused on investigating the TLR4-mediated NF-κB pathway. NF-κB is an important signal transducer in controlling transcription of DNA and cytokine production in the response to inflammation [16]. In our study, we found that Sch A could suppress the phosphorylation of NF-κB in a dose-dependent manner, block its nuclear translocation and binding to iNOS promoter gene. The activation of NF-κB is known to require regulation of upstream signal transduction such as IKKs complex formation and IκB phosphorylation and degradation [17]. We also determined the effect of Sch A on expression levels of p-IKKβ and p-IκB and found that they could be significantly decreased by Sch A at 0.5 h and 1 h, respectively. Moreover, we provided further evidence that Sch A could inhibit IKKβ and IκB phosphorylation in a concentration-dependent manner.

TRAF6 protein mediates signal transduction not only from members of the TNF receptor superfamily but also the Toll/IL-1 family. When stimulated by LPS, TRAF6 in the cytoplasm can be activated by ubiquitylation [18]. During signal transduction of the NF-κB pathway, activated TRAF6 interacts with a series of proteins, such as transforming growth factor-beta-activated kinase 1 (TAK1) and TAK1-binding proteins to form kinase complexes which can activate IKKs. Proteasome degradation of TRAF6 is a vital molecular target for the anti-inflammatory pathway [19]. A previous study demonstrated that K48-linked polyubiquitination could result in proteasomal degradation of TRAF6 [20]. In our study, we found Sch A could dose-dependently down-regulate the enhanced TRAF6 expression induced by LPS for 0.5 h.

Jak-Stats, as critical immunological signaling molecules, are normally expressed in the brain and play a vital role in neuroninflammation. Expression levels of Jak-Stats have been shown to be enhanced in reactive astrocytes and microglia cells when the brain suffers focal ischemic injury [21]. Jaks are constitutively associated with the cytoplasmic side of membrane-spanning receptors [22]. Following receptor activation by a stimulator, Jak proteins can autophosphorylate and provide a docking site for Stats. After phosphorylation by the corresponding Jak, Stats dimerize and translocate into the cell nucleus. In the nucleus, Stats initiate the control of transcription of target genes encoding acute-phase proteins as well as a number of proinflammatory cytokines and chemokines [23–24]. In our research, we found Sch A could suppress the phosphorylation levels of Jak2 and block Stat3 nuclear translocation, indicating that the Jak2-Stat3 pathway was inhibited by Sch A in LPS-induced BV-2 cells. Stat3 activation was realized by phosphorylation of Tyr705 and Ser727, two important regulatory sites. Phosphorylation at the Tyr705 site is known to play a key role during Stat3 dimer formation, nuclear translocation and DNA binding, while Ser727 phosphorylation can mediate Stat3 transcriptional activation [25]. In our study, we found that Stat3 phosphorylation at Tyr705 and Ser727 sites could be down-regulated by Sch A in a dose-dependent manner.

In order to explore the location of bio-target of Sch A, we used ciclosporin A (CsA, P-glycoprotein Pgp inhibitor) to treat BV-2 cells to block the drug efflux function of Pgp, and then we observed the inflammatory inhibitory effects of Sch A were not changed compared with non-CsA treated group (S2 Fig) [26]. These results suggested that SchA targets not intracellular proteins, because CsA treatment could not inhibit Sch A efflux or increase SchA intracellular concentration as well as its biological effects.

**Fig 7:**
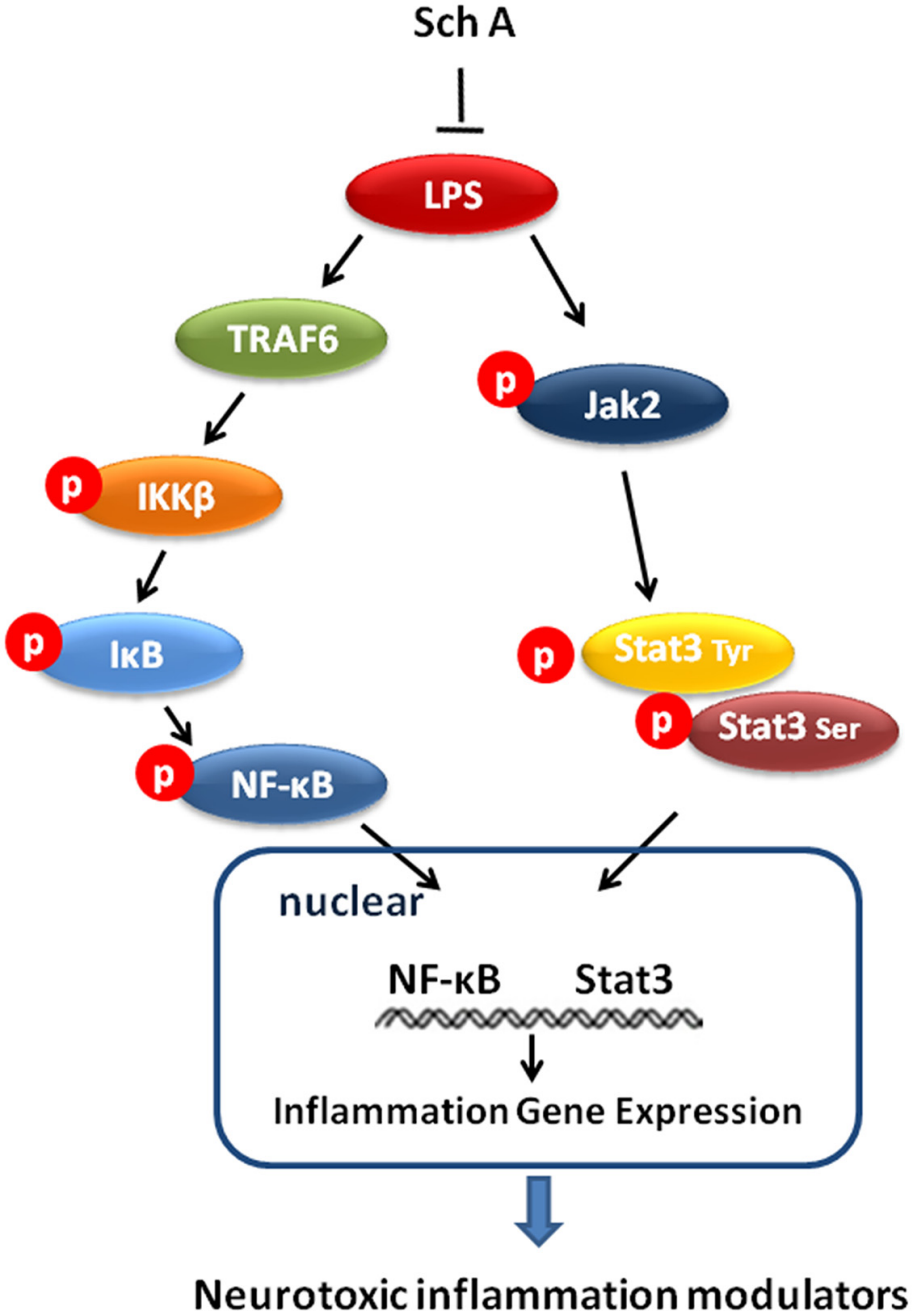
Proposed anti-neuroinflammatory mechanism of Sch A via TRAF6-NF-κB and Jak2-Stat3 pathways.

In summary, our study for the first time demonstrated that Sch A could prevent neuronal injury from microglia-mediated inflammation by decreasing the production of various inflammation mediators. The anti-inflammatory mechanisms involved interference with the TRAF6-NF-κB and Jak2-Stat3 signaling pathways (Fig 7). These data implicate Sch A as a promising drug candidate in the therapy of inflammation-related neurodegenerative diseases.

## Supporting Information

S1 Fig Sch A inhibition of NO but IL-6, TNF-α production in RAW264.7 cells induced by LPS. (A) RAW 264.7 cells were treated with LPS (1 μg/ml) with or without Sch A (10, 20 and 50 μM) for 24 h, and NO production was quantified by an assay kit. (B) RAW 264.7 cells were treated with LPS (1 μg/ml) with or without Sch A (10, 20 and 50 μM) for 8 h, followed analysis of IL-6 expression. (C) RAW 264.7 cells were treated with LPS (1 μg/ml) with or without Sch A (10, 20 and 50 μM) for 4 h, followed analysis of TNF-α expression.
(TIF)

S2 Fig Ciclosporin A (CsA) had no effect on the inhibitory effects of Sch A. BV-2 cells were treated with LPS (1μg/ml) with or without Sch A (50 μM) and CsA (5 μM) for 24 h, and then NO assay was performed.
(TIF)
