# Supplementary figures and images for: Schizandrin A Inhibits Microglia-Mediated Neuroninflammation through Inhibiting TRAF6-NF-κB and Jak2-Stat3 Signaling Pathways
Source: PLoS One. 2016 Feb 26;11(2):e0149991. doi: 10.1371/journal.pone.0149991 (PMC4768966; doi:10.1371/journal.pone.0149991)

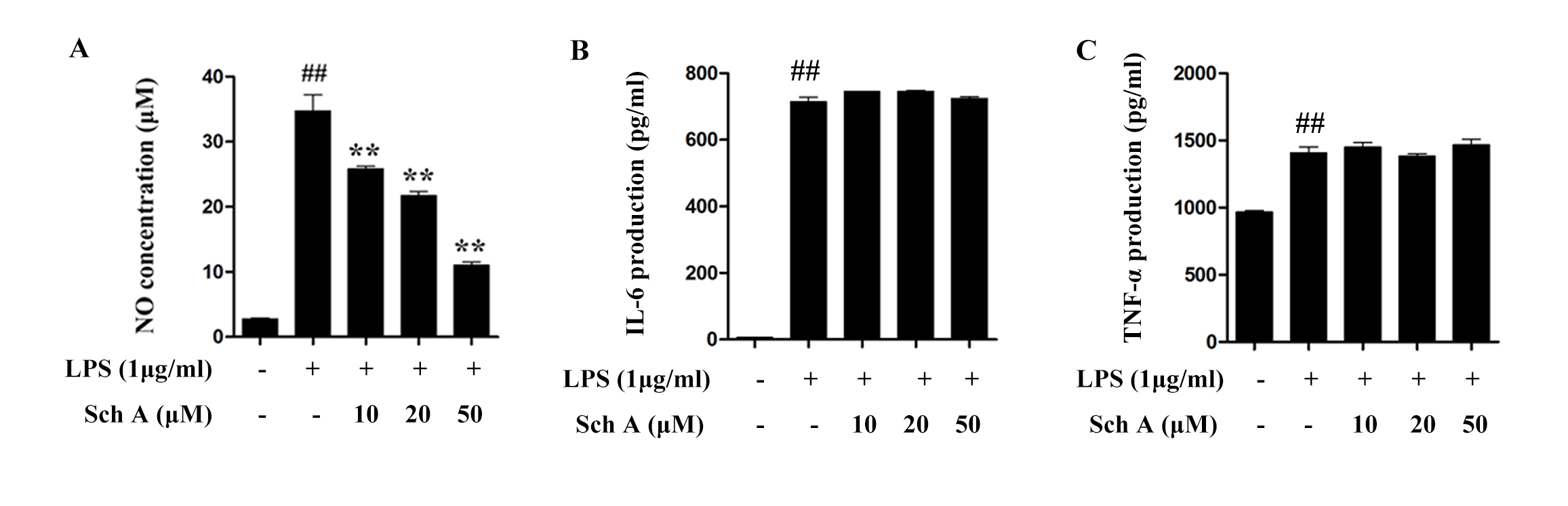

Supplement: S1 Fig — (A) RAW 264.7 cells were treated with LPS (1 μg/ml) with or without Sch A (10, 20 and 50 μM) for 24 h, and NO production was quantified by an assay kit. (B) RAW 264.7 cells were treated with LPS (1 μg/ml) with or without Sch A (10, 20 and 50 μM) for 8 h, followed analysis of IL-6 expression. (C) RAW 264.7 cells were treated with LPS (1 μg/ml) with or without Sch A (10, 20 and 50 μM) for 4 h, followed analysis of TNF-α expression. (TIF) [file pone.0149991.s001.tif]

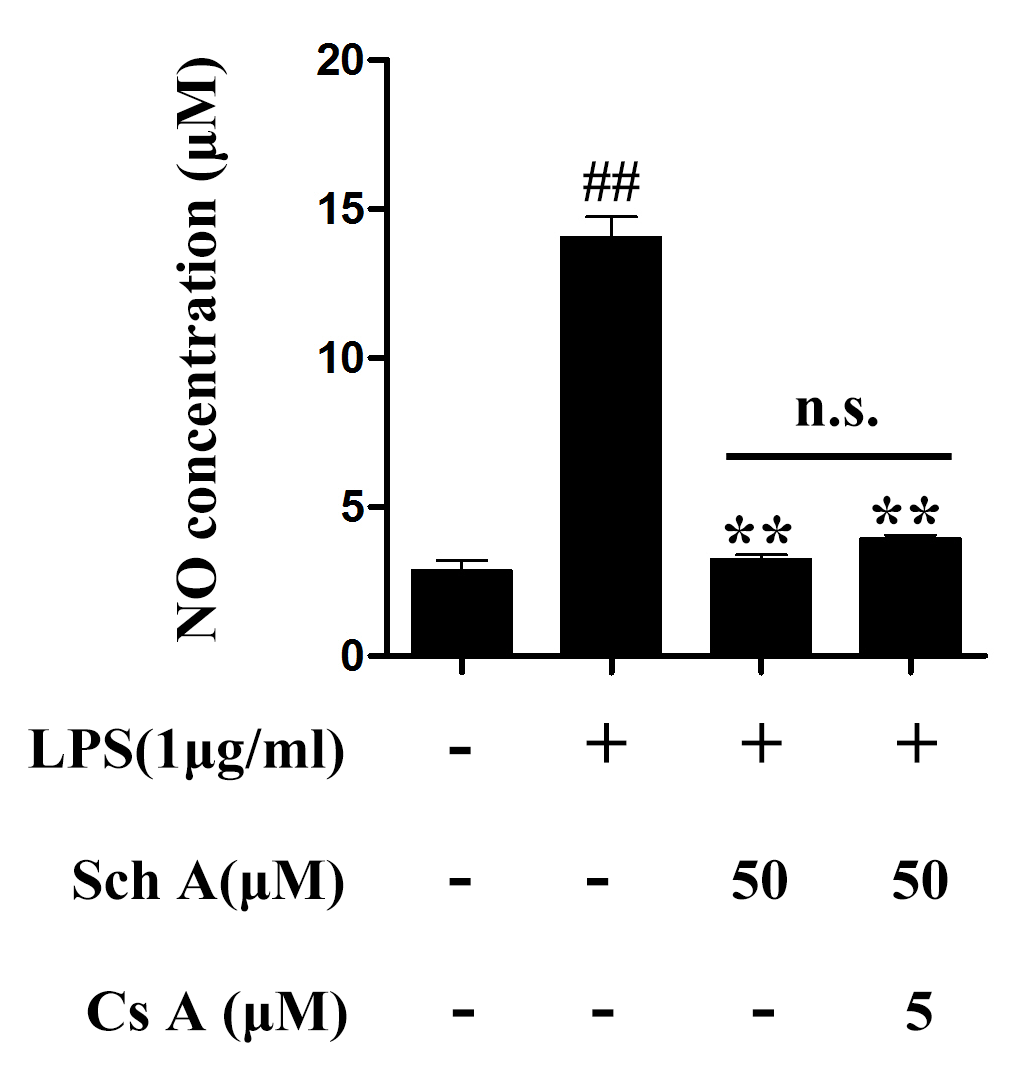

Supplement: S2 Fig — BV-2 cells were treated with LPS (1μg/ml) with or without Sch A (50 μM) and CsA (5 μM) for 24 h, and then NO assay was performed. (TIF) [file pone.0149991.s002.tif]
